# A multistate outbreak of Salmonella enterica serotype Baildon associated with domestic raw tomatoes.

**DOI:** 10.3201/eid0706.010625

**Published:** 2001

**Authors:** K Cummings, E Barrett, J C Mohle-Boetani, J T Brooks, J Farrar, T Hunt, A Fiore, K Komatsu, S B Werner, L Slutsker

**Affiliations:** Disease Investigations Section, Division of Communicable Disease Control, California Department of Health Services, Berkeley, 94704, USA. kcumming@dhs.ca.gov

## Abstract

Salmonella enterica serotype Baildon, a rare serotype, was recovered from 86 persons in eight states; 87% of illnesses began during a 3-week period ending January 9, 1999. Raw restaurant-prepared tomatoes were implicated in multiple case-control studies. Contamination likely occurred on the farm or during packing; more effective disinfection and prevention strategies are needed.


### A Multistate Outbreak of *Salmonella enterica* Serotype Baildon Associated with Domestic Raw Tomatoes

**Kate Cummings,* Elizabeth Barrett,† Janet C. Mohle-Boetani,* John T. Brooks,‡ Jeff Farrar,§ Travis Hunt,¶ Anthony Fiore,‡ Ken Komatsu,# S. Benson Werner,* and Laurence Slutsker‡**

*California Department of Health Services, Berkeley, California, USA; †Virginia Department of Health, Richmond, Virginia, USA; ‡ Centers for Disease Control and Prevention, Atlanta, Georgia, USA; §California Department of Health Services, Sacramento, California, USA; ¶United States Food and Drug Administration, Pittsburgh, Pennsylvania, USA; and #Arizona Department of Health Services, Phoenix, Arizona, USA

*Salmonella enterica* serotype Baildon, a rare serotype, was recovered from 86 persons in eight states; 87% of illnesses began during a 3-week period ending January 9, 1999. Raw restaurant-prepared tomatoes were implicated in multiple case-control studies. Contamination likely occurred on the farm or during packing; more effective disinfection and prevention strategies are needed.

We report our investigation of a large, multistate outbreak of 86 cases of salmonellosis associated with eating raw, domestic tomatoes; this is the third such outbreak in the United States in recent years (1,2).

### The Study

Outbreak patients were persons from whom *Salmonella enterica* serotype Baildon was recovered between December 1, 1998, and March 1, 1999. *S*. Baildon is rare; only five isolates were reported nationwide in 1997 (3). To increase case finding, the Centers for Disease Control and Prevention (CDC) notified epidemiologists and public health laboratorians nationwide about the outbreak.

After hypotheses-generating interviews in three states, patients from Arizona, California, Georgia, and Virginia were enrolled in four independently conducted case-control studies. Each study explored food items eaten, and place of food preparation and consumption (home vs. institution or restaurant), for the 5 to 7 days before illness began. Controls were matched to patients by gender, age, geographic area, and case-specific exposure period. Ten Arizona patients were compared with 18 controls identified by systematic telephone digit-dialing. Seventeen California patients were compared with 32 healthy controls previously infected with nontyphoidal *Salmonella*; five Georgia patients were compared with 10 controls identified by patients as friends; 11 Virginia patients were compared with 33 controls drawn from a systematic sample of reverse telephone directories.

The distribution sources of tomatoes for 15 tomato point of service (POS) exposures reported by 14 patients in California and Virginia were examined. POS included three Virginia and two California restaurants, six outlets of one Mexican fast-food restaurant chain in California, and two Virginia nursing homes. Tomato operations were observed at one grower/packer cooperative, five Virginia facilities, and the sole-source processor of diced tomatoes used by the Mexican restaurant chain in California.

We calculated Mantel-Haenszel matched odds ratios and p values to assess univariate associations between food items and illness. Three of four case-control studies suggested two food items. Using data collected from the California case-control study, we assessed the independent association of these two food items by conditional logistic regression.

We identified 86 patients from eight states (Table). Onset dates were from December 6, 1998 to February 2, 1999; 87% occurred in a 3-week period ending January 9, 1999. Three elderly persons died.

Arizona patients were significantly more likely than controls to report eating at a specific chain of Mexican fast-food restaurants (60% vs. 13%, matched odds ratio (MOR) undefined, $p = 0.008$) but no food item was implicated. California patients were significantly more likely than controls to report eating five restaurant-prepared foods: raw tomatoes (94% vs. 33%, MOR 20, $p = 0.002$), iceberg lettuce (88% vs. 40%, MOR 16.5, $p = 0.008$), cheese (88% vs. 43%, MOR 6.6, $p=0.02$), raw onions (77% vs. 16%, MOR 10.3, $p<0.001$) and sour cream (76% vs. 11%, MOR 21.5, $p <0.001$). California patients were also more likely than controls to report eating at the same chain of Mexican fast-food restaurants identified in the Arizona study (63% vs. 17%, MOR 14.5, $p = 0.01$). There was a trend toward an association with eating home-prepared raw tomatoes (81% vs. 48%, MOR 6.0, $p = 0.08$). In a regression model containing both restaurant-prepared tomatoes and iceberg lettuce, tomatoes but not iceberg lettuce remained associated with disease (maximum likelihood estimate [MLE] 11.2, Wald $p = .08$ vs. MLE 1.6, Wald $p = 0.74$).

Address for correspondence: Kate Cummings, Disease Investigations Section Division of Communicable Disease Control, 2151 Berkeley Way, Room 708, Berkeley, CA 94704, USA; fax: 510-540-2570; e-mail: kcumming@dhs.ca.gov

Table. Characteristics of patients with culture-confirmed *Salmonella enterica* Serotype Baildon

| State | Cases (#) | Hospitalized[a] (#) | Deaths[a] (#) | Median age (years) | Age range (years) | Patients ≥18 years of age (%) | Female (%) | Range of onset dates[b] |
|---|---|---|---|---|---|---|---|---|
| CA | 44 | 11 | 1 | 33 | <1-82 | 89 | 65 | 12/18/98-02/02/99 |
| VA | 13 | 4 | 1 | 47 | 20-86 | 100 | 69 | 12/21/98-01/09/99 |
| AZ | 13 | 0 | 0 | 26 | 18-69 | 92 | 69 | 12/18/98-01/29/99 |
| GA | 8 | 1 | 1 | 38 | 17-86 | 88 | 75 | 12/19/98-02/02/99 |
| IL | 3 | 0 | 0 | 43 | 32-58 | 100 | 33 | 12/23/98-01/07/99 |
| AL | 2 | 0 | 0 | 66 | 45-86 | 100 | 100 | 01/07/99 |
| TN | 2 | 0 | 0 | 46 | 41-51 | 100 | 50 | 01/04/99-01/06/99 |
| KS | 1 | 0 | 0 | 22 | 22 | 100 | 100 | 12/06/98 |
| Total | 86 | 16 | 3 | 35 | <1-86 | 93 | 67 | 12/06/98-02/02/99 |

[a]Data available for 91% (78) of patients.

[b]Specimen collection date was used if patient's symptom onset date was unknown (n = 10)

Georgia patients demonstrated a trend toward eating restaurant-prepared tomatoes (80% vs. 20%, MOR 8.7, p = 0.09) and iceberg lettuce (100% vs. 60%, MOR undefined, p = 0.10). Virginia patients were significantly more likely than controls to report eating institution- or restaurant-prepared raw tomatoes (91% vs. 45%, MOR 11.1, p = 0.009) and cucumbers (73% vs. 33%, MOR 5.4, p = 0.03). Patients demonstrated a trend toward eating restaurant-prepared iceberg lettuce (73% vs. 52%, MOR 2.2, p = 0.21), raw onion (55% vs. 27%, MOR 2.9, p = 0.1), and romaine lettuce (36% vs. 9%, MOR 4, p = 0.07).

The traceback identified two tomato grower/packer cooperatives, in Florida, which could have supplied tomatoes eaten by the 14 patients who reported only one or two POS encounters during the exposure period. In April 1999, the only cooperative still packing tomatoes was investigated. Tomatoes had reportedly been hand-picked and were transported to the packing facilities in covered bins. Tomatoes were unloaded into a dump tank and moved by a flume system (water temperature 38.7°C, pH 6.5, target chlorine reported as 125 ppm but not measured) to a warm spray wash. Tomatoes were mechanically sorted (unacceptable tomatoes were manually removed), waxed, and boxed. Packed tomatoes were stored at 21.1°C in ripening rooms.

The tomato dicing operation in California was inspected in May 1999. Uncored tomatoes were washed, inspected for decay, color, and stem removal, and then conveyed to a mechanical dicer. Diced tomatoes were moved by a flume system to a perforated shaker-belt conveyor, mechanically packaged into 5-pound trays, sealed and stored at 4.4°C. Tomatoes were held for one day before being shipped by refrigerated truck to two distributors. Target water temperature, total chlorine, and hold-times for the bath and flume were reported by the processor as 1.1°C, 100-130 ppm, and 1-2 minutes, respectively. Wash water temperatures and chlorine levels were maintained manually whereas the flume system was chlorinated by an automated system. During inspection, this system's pH monitor did not work. Temperature was measured at 2.20°C.

Tomatoes served in Virginia were processed at the individual POS facilities. Whole, uncored tomatoes were washed and cut by knife or mechanical chopper.

### Conclusions

We report on a large, multistate outbreak caused by *S.* Baildon, an unusual *Salmonella* serotype. The outbreak was associated with eating raw tomatoes. Because less than three percent of estimated *Salmonella* cases are officially reported nationwide (4,5), this outbreak could have included 3,300 cases.

Raw tomatoes were epidemiologically implicated as the source of this outbreak. This finding is supported by several observations. First, eating raw tomatoes was strongly associated with illness in the case-control studies, and nearly all patients ate them. Second, these studies were conducted independently, using different control recruitment strategies. Third, raw tomatoes have a 3-week shelf life, consistent with the brief occurrence of the outbreak.

That many restaurants across several states were involved suggests the tomatoes were likely contaminated early on—at the farm or during packing. Salmonellae can grow on tomato skin surfaces and infiltrate core tissues during tomato harvest, packing, and transportation (6,7). Air spaces in tomatoes at high field-heat temperatures can constrict when submerged in cool water. As air space volume decreases, water and salmonellae can be drawn (by vacuum effect) from the dump tank into the fruit through the stem scar. For these reasons, postharvest process water should be potable and warmer than the incoming fruit (8).

Once tomatoes are contaminated, elimination of salmonellae can be difficult. While chlorine levels of 200-250 ppm would be expected to substantially reduce salmonellae (6,7), even higher levels of chlorine disinfection (320 ppm) did not eliminate salmonellae from tomatoes in one laboratory study (6). The efficacy of chlorine against salmonellae depends, in part, on the location and amount of contamination. Salmonellae inoculated onto stem scars and growth cracks survived disinfection better than on smooth tomato skins (7).

The grower/packer cooperative we observed had at least some elements of a hazard analysis critical control point (HACCP) program for commercial tomato packinghouses (9) including warm, chlorinated wash water. However, we observed operations after the outbreak and did not have access to historic water quality measures (free chlorine, pH, and temperature). Even if free chlorine levels of 125 ppm were maintained, such levels would not be expected to eliminate organisms in stem scars or damaged tomato skin.

Dicing and pooling of contaminated tomatoes in our outbreak may have played a role in amplifying the amount of contaminated product, just as these were suspected to have played a role in prior outbreaks (2). The diced tomato processor we observed in California exposed both whole and diced tomatoes to chlorine. However, laboratory experiments demonstrated that *S*. Baildon could survive disinfection with 200 ppm chlorine in diced tomatoes (10). Microorganisms in tomatoes are highest around the stem scar and central core (11), where they are less accessible to chlorine (7). Therefore, the practice of including stem scars and cores in pooled, finished product could have increased the opportunity for amplification, especially if the diced tomatoes were later mishandled. Contamination of internal tissue from the outer skin and stem scar can also occur during cutting and slicing (12). Numerous *Salmonella* serotypes, including our outbreak strain, grow rapidly in cut tomatoes held at room temperature (6,7,10,13). If the involved restaurants maintained tomatoes at room temperature for extended periods, even small populations of salmonellae on sliced or diced tomatoes could have grown rapidly.

While chlorine-based water quality systems may markedly reduce salmonellae contamination, they cannot be relied upon to eliminate it. A terminal treatment step with demonstrated effectiveness against *Salmonella*, such as irradiation (14,15), should be considered, particularly since tomatoes are commonly eaten raw and have now been implicated in three multistate outbreaks.

### Acknowledgments

We thank Clare Kioski, Sharon Abbott, Michael Gutierrez, Robert Murray, Patricia Griffin, Adriana Lopez, Jane Koehler, Susan Lance-Parker, Connie Austin, Carl Langkop, Gail Hansen, Craig Allen, Mary Ellen Chesser, Robert Taylor, Pete Cook, Dennis Doupnik, John Guzewich, Ellen Morrison, Ryan Davis, Christopher Thackston, Karen Chaples, Robert Hackler, Kathy Highfill, Anna Kennedy, Charles Kennedy, Tim LaFountain, Martha Lees, Michael Leffue, Tim Myers, Molly O'Dell, Steve Shepard, Mary Tinsley, Diane Woolard, Jackie Zeltvay, Adele Zmarzly, Judith Carroll, Kathryn Dupnik, Sarah Henderson, Mary Mismas, Daksha Patel, Tanea Reed, and Denise Toney.

Ms. Cummings is an epidemiologist in the California Department of Health Services; her work focuses on the epidemiology of infectious diseases in California.

### References

1. Beuchat LR. Surface decontamination of fruits and vegetables eaten raw: a review. Geneva: World Health Organization; 1998 WHO/FSF/FOS/98.2.
2. Hedberg CW, Angulo FJ, White KE, Langkop CW, Schell WL, Stobierski MG, et al. Outbreaks of salmonellosis associated with eating uncooked tomatoes: implications for public health. Epidemiol Infect 1999;122:385-93.
3. Centers for Disease Control and Prevention. *Salmonella* surveillance: annual tabulation summary 1997. Atlanta: The Centers; 1998.
4. Mead PS, Slutsker L, Dietz V, McCaig LF, Bresee JS, Shapiro C, et al. Food-related illness and death in the United States. Emerg Infect Dis 1999;5:607-25.
5. Chalker RB, Blaser MJ. A review of human salmonellosis: III. Magnitude of *Salmonella* infection in the United States. Rev Infect Dis 1988;9:111-24.
6. Zhuang RY, Beuchat LR, Angulo FJ. Fate of *Salmonella montevideo* on and in raw tomatoes as affected by temperature and treatment with chlorine. Appl Environ Microbiol 1995;61:2127-31.
7. Wei CI, Huang TS, Kim JM, Lin WF, Tamplin ML, Bartz JA. Growth and survival of *Salmonella montevideo* on tomatoes and disinfections with chlorinated water. J Food Protect 1995;58:829-36.
8. U.S. Food and Drug Administration, U.S. Department of Agriculture, and Centers for Disease Control and Prevention. Guidance for industry: guide to minimize microbial food safety hazards for fresh fruits and vegetables. Washington: Center for Food Safety and Applied Nutrition, Food and Drug Administration; 1998. Available at URL: http://www.foodsafety.gov/~dms/prodguid.html
9. Rushing JW, Angulo FJ, Beuchat LR. Implementation of a HACCP program in a commercial fresh-market tomato packinghouse: a model for the industry. Dairy, Food and Environmental Sanitation 1996:16:549-53.
10. Weissinger WR, Chantarapanont W, Beuchat LR. Survival and growth *of Salmonella baildon* in shredded lettuce and diced tomatoes, and effectiveness of chlorine as a sanitizer. Int J Food Microbiol 2000;62:123-51.
11. Samish Z, Etinger-Tulczynska. Distribution of bacteria within the tissue of healthy tomatoes. Appl Microbiol 1963;11:7-10.
12. Lin CM, Wei CI. Transfer of *Salmonella montevideo* onto the interior surfaces of tomatoes by cutting. J Food Protect 1997;60:858-63.
13. Asplund K, Nurmi E. The growth of salmonellae in tomatoes. Int J Food Microbiol 1991;13:177-82.
14. Wood OB, Bruhn CM. Position of the American Dietetic Association: food irradiation. J Am Diet Assoc 2000;100:246-53.
15. Monk JD, Beuchat LR, Doyle MP. Irradiation inactivation of food-borne microorganisms. J Food Protect 1995;58:197-208.

## References

[PDF_00294] Asplund K., Nurmi E. (1991). The growth of salmonellae in tomatoes.. Int J Food Microbiol.

[PDF_00263] Chalker R. B., Blaser M. J. (1988). A review of human salmonellosis: III. Magnitude of Salmonella infection in the United States.. Rev Infect Dis.

[PDF_00253] Hedberg C. W., Angulo F. J., White K. E., Langkop C. W., Schell W. L., Stobierski M. G., Schuchat A., Besser J. M., Dietrich S., Helsel L. (1999). Outbreaks of salmonellosis associated with eating uncooked tomatoes: implications for public health. The Investigation Team.. Epidemiol Infect.

[PDF_00260] Mead P. S., Slutsker L., Dietz V., McCaig L. F., Bresee J. S., Shapiro C., Griffin P. M., Tauxe R. V. (1999). Food-related illness and death in the United States.. Emerg Infect Dis.

[PDF_00289] SAMISH Z., ETINGER-TULCZYNSKA R. (1963). Distribution of bacteria within the tissue of healthy tomatoes.. Appl Microbiol.

[PDF_00285] Weissinger W. R., Chantarapanont W., Beuchat L. R. (2000). Survival and growth of Salmonella baildon in shredded lettuce and diced tomatoes, and effectiveness of chlorinated water as a sanitizer.. Int J Food Microbiol.

[PDF_00296] Wood O. B., Bruhn C. M. (2000). Position of the American Dietetic Association: food irradiation.. J Am Diet Assoc.

[PDF_00266] Zhuang R. Y., Beuchat L. R., Angulo F. J. (1995). Fate of Salmonella montevideo on and in raw tomatoes as affected by temperature and treatment with chlorine.. Appl Environ Microbiol.

